# Mechanisms of Adaptive Immunity to Porcine Reproductive and Respiratory Syndrome Virus

**DOI:** 10.3390/v9060148

**Published:** 2017-06-13

**Authors:** Michael C. Rahe, Michael P. Murtaugh

**Affiliations:** Department of Veterinary and Biomedical Sciences, University of Minnesota, 1971 Commonwealth Avenue, St. Paul, MN 55108, USA; murta001@umn.edu

**Keywords:** PRRSV, T cell, B cell, NK cell, neutralizing antibody, porcine, memory, adaptive immune response

## Abstract

The adaptive immune response is necessary for the development of protective immunity against infectious diseases. Porcine reproductive and respiratory syndrome virus (PRRSV), a genetically heterogeneous and rapidly evolving RNA virus, is the most burdensome pathogen of swine health and wellbeing worldwide. Viral infection induces antigen-specific immunity that ultimately clears the infection. However, the resulting immune memory, induced by virulent or attenuated vaccine viruses, is inconsistently protective against diverse viral strains. The immunological mechanisms by which primary and memory protection are generated and used are not well understood. Here, we summarize current knowledge regarding cellular and humoral components of the adaptive immune response to PRRSV infection that mediate primary and memory immune protection against viruses.

## 1. Introduction

Porcine reproductive and respiratory syndrome virus (PRRSV) is the most severe enemy of porcine health and wellbeing. The highly mutable, enveloped, RNA virus was discovered nearly 30 years ago but, while extensive research has been carried out and many vaccines have been developed, there is still no reproducible immunological intervention that develops a broadly protective immune response against virulent PRRSV. 

PRRS disease was first described on farms in North Carolina in the USA at the end of the 1980s. Outbreaks were marked by reproductive losses, post-weaning pneumonia, and increased mortality in growing pigs. Initial efforts to identify an etiological agent responsible for the new disease syndrome were unsuccessful, leading to the disease being temporarily designated mystery swine disease (MSD) in North America. Koch’s postulates for MSD were fulfilled in 1991 with a previously unidentified RNA virus discovered in Europe, named Lelystad virus [1,2]. The discovery was quickly followed by isolation of the virus, initially referred to as swine infertility and respiratory syndrome virus or SIRS virus, in North America [3]. 

The name PRRSV was introduced in 1992 and encompasses PRRSV-1 (genotypes first isolated in Europe) and PRRSV-2 (genotypes first isolated in North America) [4,5]. Today, both virus types are globally distributed, with PRRSV-1 viruses predominantly in Europe and PRRSV-2 viruses largely in North America, Asia and South America [6]. Recent discovery of multiple arteriviral nucleotide sequences in nonhuman primates has led to a reclassification of PRRSV as two distinct viruses, PRRSV-1 and PRRSV-2 [7]. Here, we use the generic PRRSV to refer broadly to both viruses when evidence indicates that are equivalent, and the specific PRRSV-1 and PRRSV-2 is used when a distinction is desired. The reasoning is based on the many similarities of the two viruses in fine details of genome structure and organization, transcriptional strategy, host preference, clinical signs of disease, and anti-viral immunity [7,8,9,10,11]. In particular, chimeric PRRSV consisting of PRRSV-1 open reading frames (ORFs) 2–5 in a background of PRRSV-2 are fully viable, showing as well that the molecular signals for transcription and translation are preserved [12].

PRRSV has a positive-sense, single-stranded RNA genome of approximately 15 kb designated to the *Arteriviridae* family. The virus encodes at least 10 functional ORFs. ORF1a and 1b encode two large polyproteins which are cleaved into 14 non-structural proteins [13]. There are eight known structural proteins encoded by ORF2a, ORF2b, ORF3–7 and ORF5a [14,15,16]. PRRSV is one of the most rapidly mutating RNA viruses known, with considerable genetic variation within both PRRSV-1 and PRRSV-2, based on ORF5 phylogenetic analysis [10,17]. This impressive genetic diversity makes the development of a broadly protective immune response to vaccination difficult to achieve. After infection, the virus can endure and replicate in the host, depending on immune status and PRRSV strain, for a period of at least 150 days [18]. Therefore, contrary to being labeled repeatedly as a persistent pathogen, animals are capable of eventually clearing PRRSV. However, the components of the immune system responsible for the development of sterilizing immunity are not completely understood or have yet to be discovered. Here, we will discuss several aspects of PRRSV antigen-specific and protective immunity which have yet to be elucidated while focusing on potential areas of further investigation. Readers interested in additional reviews of PRRSV literature related to immunity are directed to the following articles [11,19]. 

## 2. The Targets of Infection

PRRSV infects cells of the macrophage/monocyte lineage, including dendritic cells [20,21,22,23]. Permissive cells express Cluster of Differentiation (CD)163, a hemoglobin-haptoglobin scavenger, which is the necessary receptor for PRRSV infection and replication [24,25,26]. Macrophages and dendritic cells are common members of the mononuclear phagocyte system that plays a varied, and important, role in many aspects of tissue remodeling, development, immunity and immunopathology [27]. Classically designated as part of the innate immune system, these leukocytes are critical for the development of a productive adaptive immune response. Macrophages and, particularly, dendritic cells take up and present antigen to T cells and B cells, thus initiating an adaptive immune response against the presented antigen [28,29]. If a pathogen is able to infect and destroy, manipulate, or maintain itself within macrophages or dendritic cells, it then has the potential to modulate the immune response into a favorable situation for its own replication and survival. 

Therefore, many pathogens employ strategies for macrophage infection as a way to make the host more amenable to infection. Recent research into *Mycobacterium tuberculosis* (Mtb) has shown that, after phagocytosis, the bacterium arrests phagosome maturation and intra-phagosome lipolysis resulting in Mtb survival and an increased supply of nutrients for growth [30,31]. Human immunodeficiency virus (HIV) infects macrophages to establish reservoirs within the host for the chronic stage of the disease when CD4^+^ T cells are largely depleted and neutralizing antibodies may be present [32,33,34]. *Leishmania major* is a protozoan which infects phagocytes to subvert the immune system. The parasite expresses glycoprotein (gp)63, a multifaceted surface-expressed pathogenicity factor that is responsible for preventing antigen presentation and killing by natural killer (NK) cells [35,36,37]. Indeed, there are many more examples of burdensome pathogens which target phagocytic cells, especially macrophages and dendritic cells, in an attempt to gain a foothold within the immune system and allow for unchecked survival and replication [38,39,40]. PRRSV is one of these pathogens.

The ability of PRRSV to subvert the immune system has not been investigated as extensively as more prominent pathogens of humans, such as HIV. PRRSV has been shown to inhibit the production, or the downstream effects, of type 1 interferons, particularly interferon (IFN)-α, on intracellular signaling [41,42,43,44,45,46,47,48]. Interestingly, multiple PRRSV proteins (nonstructural protein (nsp) 1, nsp2, nsp4, nsp5, nsp11 and nucleocapsid) have been reported to possess interferon inhibiting abilities. 

In addition, a number of in vivo experiments have reproduced earlier in vitro findings showing that interferon-α is inhibited during the early stages of PRRSV infection [47,49,50]. While the impact of type 1 interferon suppression is likely to create a favorable environment for the virus to replicate and survive in phagocytic cells, it is still unclear what effect, if any, suppression of type 1 interferon activity has on the adaptive immune response to infection [51]. Future investigations could clarify the relative contributions of viral proteins on modulation of interferon production and their impacts on viral growth, survival, and the subsequent development of the adaptive immune response. 

Apart from interfering with interferon expression, PRRSV has also displayed the in vitro ability to subvert the immune system by spreading from cell to cell. Recent work has uncovered the ability of the virus to spread infectious viral RNA, several replicases, and certain structural proteins between cells via intercellular nanotubules [52,53]. While this activity theoretically allows for PRRSV to avoid neutralizing antibodies, the presence and significance of this mechanism in PRRSV pathogenesis has yet to be fully elucidated. Future studies are needed to determine if this process operates in naturally permissive macrophages and dendritic cells, if it can be interrupted, for example by intracellular antibodies, and what effect it might have on viral propagation [54,55].

Vaccines depend upon innate immune stimulation to promote effective adaptive immune response to antigen, resulting in production of antibodies and cytotoxic T cell responses. The ability of a pathogen to successfully infect and replicate within innate immune cells makes the development of a protective immune response more difficult. As a result, the generation of effective vaccines against pathogens that target immune cells is fraught with challenges. Extensive variation in viral genetics, primary immune responses, and cross-protection indicates that much remains to be learned about cellular pathogenesis in order to arrive at better immunological solutions. 

## 3. Immunosuppression

Immunosuppression refers to suppression of the immune system and its ability to fight infection. HIV and infectious bursal disease virus are examples of viral infections that destroy entire lymphoid cell populations that ablate or disable adaptive immune responses. Lymphoproliferative cancers block cellular differentiation and deprive the body of mature, effector lymphocytes, thus causing immunosuppression in a different manner. PRRSV does neither; infection does not lead to severe lymphoid depletion or ablation, and it does not interfere profoundly with lymphocyte differentiation or maturation. Leukocyte perturbations in lymphoid tissues are associated with PRRSV infection, suggesting that adaptive immunity might be weakened, though not destroyed [56,57,58,59,60,61]. 

The immune system also maintains peripheral tolerance to self and commensal bacteria through immunosuppressive mechanisms that include regulatory T cells (Tregs), characterized as CD4^+^CD25^+^Forkhead box p3 (Foxp3)^+^ T lymphocytes [62]. Treg suppressive properties were discovered when thymectomized or Treg-depleted mice succumbed to autoimmune reactions [63,64]. Tregs suppress effector and effector memory T cell proliferation by cytokine deprivation leading to polyclonal apoptosis, and by suppression of antigen presenting cells by cytotoxic T lymphocyte-associated antigen-4 (CTLA-4) and other mechanisms [62]. Studies in PRRSV infections give an ambiguous picture about the role of Tregs. PRRSV-2 strains are reported to induce a strong Treg response which included transforming growth factor (TGF)β-1 secretion in vitro as well as in vivo [65,66]. Other studies did not show Treg responses to infection with either PRRSV-1 or PRRSV-2 [67,68]. Interleukin-10 (IL-10), an immunosuppressive cytokine expressed by various cell types including Tregs, was induced by PRRSV-2 vaccination in weaned pigs in one study, but was not induced in weaned or adult pigs in another study [69]. Additional in vitro and in vivo studies reported IL-10 mRNA transcription and cytokine production after PRRSV infection [70,71,72]. However, kinetic analysis in serum of viremic pigs of various ages showed that elevated IL-10 levels were primarily a function of age and were not associated with infection status [69]. The only exception was in weaned pigs infected with a virulent virus, in which a transient increase was associated with viral pathogenesis [69]. 

On balance, the immunological evidence for PRRSV inducing a state of immunosuppression does not appear to be compelling. Secondary infections following PRRS disease outbreak in swine herds, suggesting a reduced ability to fight infection, is an alternative indicator of immunosuppression. An early study showed concurrent pulmonary bacterial infections in 58% of 221 PRRS cases [73]. However, the study did not determine if bacterial infections were present before the PRRS outbreaks. The immunosuppression question also was addressed in more controlled settings using dual infection models with PRRSV and various bacterial species. A summary of published literature in 2003 showed no predisposition to bacterial disease in 8 of 15 coinfection models, three ambiguous outcomes, and four cases in which severity of disease was increased [74]. More recent studies found a positive association between PRRSV infection and replication of porcine circovirus 2 (PCV2) or swine influenza virus [75,76].

It is possible that bacterial infections in swine herds increase following PRRS outbreaks due an increased burden of viral infection on host resilience to pathogen burden. Subclinical viral and bacterial infections are common, with PCV2, *Salmonella enterica*, *Haemophilus parasuis*, various *Mycoplasma* species, *Leptospira*, and *Escherichia coli* being examples. Control of infection is maintained by a combination of immune resistance to microbial replication and tissue tolerance to damage. In a coinfection model of influenza virus and *Legionella pneumophila*, it was clearly demonstrated that *L. pneumophila* infection was subclinical in healthy mice, but was lethal in the presence of influenza virus [77]. Overwhelming disease was due to loss of tissue resilience, since the bacterial load was unchanged [77]. This model might account for mortalities observed in experimental swine following PRRSV exposure [78]. Given the variable results of PRRSV coinfection models in swine and an alternative mechanism for increased disease in PRRSV-infected herds, generalized immunosuppression does not appear to be a key feature of PRRSV pathogenesis.

PRRSV, like many viruses, has developed countermeasures to host immune responses that enable it to survive and replicate for extended periods of time before the infection is resolved. PRRSV modulation of intracellular antiviral defense mechanisms has been reviewed extensively [79]. The effects of PRRSV infection on adaptive immune response, i.e., antigen-specific T cell, B cell, and antibody responses, are less well characterized. The antiviral response of T cells to PRRSV, examined primarily by the IFNγ enzyme-linked immunospot (ELISPOT), appears to develop slowly over a period of weeks, and is not associated with changes in viral loads in blood or in infected lung and lymphoid tissues [80,81]. Peripheral blood mononuclear cells (PBMC) from young, weaned pigs show limited IFNγ responses even when stimulated by phytohemagluttinin, which might account for the low anti-PRRSV responsiveness after re-stimulation in vitro [69]. However, PBMC from growing pigs and mature sows, which showed higher levels of IFNγ sensitivity, still showed limited responsiveness [69]. These findings indicate that PRRSV may interfere with specific cell-mediated immunity, but more direct evidence is needed for a fuller understanding.

By contrast, the interaction of PRRSV with pigs does not appear to retard or attenuate the development of humoral immunity or B cell differentiation. Induction of antibody responses to PRRSV proteins, both structural and non-structural, occurred in the same time frame as antibody responses to keyhole limpet hemocyanin (KLH), an irrelevant protein antigen [51]. The antibody response to KLH was also the same in the presence or absence of PRRSV infection [51]. Similarly, PRRSV infection did not inhibit cellular or humoral immune protection in response to pseudorabies virus vaccination [82]. Thus, the adaptive B cell response is not delayed or suppressed by PRRSV. 

An extended viremia and prolonged survival in lymphoid tissues is characteristic of PRRSV infection. These features show that PRRSV has mechanisms of immune avoidance that are not present in viruses such as influenza virus and foot and mouth disease virus, in which sterilizing immunity is achieved within 10–14 days. It appears from the findings of field observations and experimental investigations that some type of PRRSV-specific T cell interference is present, whereas specific B cell inhibition or a generalized state of immunosuppression are not immunological hallmarks of PRRSV infection.

## 4. Antibody Response

### 4.1. Neutralizing Antibody Response

The antibody response to PRRSV typically dominates discussions of PRRSV immunity, as neutralizing antibodies are the crucial component of immune-mediated protection against most viral infections [83,84]. As a result, shortly after the identification of PRRSV as the causative agent of Mystery Swine Disease, there was a strong push to identify the presence and dynamic response of neutralizing antibodies against PRRSV and then to characterize their specificity for PRRSV variants. Early work suggested that neutralizing antibodies against homologous PRRSV could be found as early as 9–11 days after inoculation [85]. However, this was likely the non-affinity matured immunoglobulin (Ig)M response, as anti-swine IgM ablated the previously observed neutralizing activity. Subsequent research showed that the high affinity neutralizing IgG response, detected at around 28–42 days post-inoculation, is specific for the inoculating virus with partial neutralizing activity against heterologous viruses [86,87,88,89,90]. 

Following the identification of PRRSV neutralizing antibodies, the effectiveness of immunoglobulins in protecting against infection was evaluated with passive transfer studies. These experiments displayed the effectiveness of neutralizing antibodies at preventing clinical infection and disease against homologous challenge [91,92]. However, these studies also showed that immune protection can be quite limited, especially between PRRSV-1 and PRRSV-2 [93]. Within PRRSV-1 or PRRSV-2, protection against homologous inoculation is consistently solid, whereas protection against heterologous challenge is variable for unclear reasons [93,94,95]. However, genetic similarity, based primarily on ORF5 sequence comparisons, shows no relationship with degree of protection [96]. These results appeared to explain the potential field problem, in which vaccinated or live virus inoculated animals become infected with a variant PRRSV genetically different enough from the inoculating strain to evade the immune system, propagate, and then cause disease. Hence, ever since the mutability, antigenic variability, and resultant immunological elusiveness of PRRSV were first appreciated, a broadly neutralizing antibody response to PRRSV has been coveted by immunologists and practitioners [97]. 

Recent research shows that there are animals capable of developing a broadly neutralizing antibody response to genetically disparate viruses [9,98]. However, this immune capability has only been found in a proportion of animals in groups of similar genetics age, sex, and exposure history [9]. The seemingly random ability of some animals to develop broadly neutralizing antibodies suggests that the inherent variation of the adaptive immune response may play a role in conferring broadly neutralizing capabilities to certain animals. Investigations into this ability are needed at the lymphocyte level and while the obvious target is the B cell, T cells cannot be overlooked, as the induction of a humoral immune response requires antigen-specific T cell driven help [99,100]. Therefore, animals able to develop a strong neutralizing antibody response would require both B cells and T cells that are capable of recognizing neutralizing epitopes. 

The conditions needed to achieve cross-neutralizing antibody production are not known, but may involve multiple exposures to the same or different virus isolates. Sows with high titered, broadly neutralizing antibodies were found in herds with multiple exposures to virulent field viruses [9]. In an experimental study, cross-neutralization was reported in animals exposed first to a PRRSV vaccine strain followed by homologous or heterologous virus challenge [86]. However, the majority of data analyzed were below the neutralization assay cutoff. Duration of viremia, up to 42 days, was linked with increased breadth of neutralizing antibodies following a single viral infection [101]. However, since cross-neutralization activity and titer data were not presented, it was not possible to further interpret the results. The animals were not subsequently challenged, so it is not known if the cross-neutralizing activity in serum was predictive of protection. Other studies showed that significant neutralizing antibody responses are not commonly observed during viremic infection of young pigs, as well as in adult sows [69,102,103,104]. 

Recently, vaccinology research in HIV has shown that sequential immunizations, tailored for specific stages of the immune response, may be useful for inducing broadly neutralizing antibodies [105,106,107]. The approach is based on the finding that early immune responses to HIV resulted in neutralizing antibodies against the circulating virus which quickly led to immune escape of the virus and the ineffectiveness of generated antibodies. The antibody-resistant virus then stimulated a secondary antibody response which again selected for antibody resistant virus. This virus-antibody hide and seek continued, eventually resulting in the selection of several neutralization targets of the virus as well as the generation of broadly neutralizing antibodies [108,109,110]. Cloning of the antibodies showed that somatic mutations are generally necessary for antibody neutralizing capabilities against HIV-1 [111,112]. These findings have shown that the B cell response of the host adapts in the germinal center as the virus evolves, suggesting that tailored sequential immunization could lead to the development of a broadly neutralizing antibody response [113]. 

The consistent generation of a broadly neutralizing antibody response to PRRSV on the herd level has evaded the swine health industry since the emergence of PRRSV. There are multiple proposed mechanisms by which PRRSV may evade or inhibit the development, or the effectiveness, of a neutralizing antibody response, such as glycan shielding of envelope glycoprotein (GP)3 or GP5 [114,115], the existence of decoy epitopes in GP5 [116], lymphocyte dysregulation [79], and inhibition of the innate immune response [117]. Comprehension of defense mechanisms employed by PRRSV makes the development of a broadly neutralizing immune response appear to be a daunting task. However, as previously shown, some animals are capable of developing such a response. Simply, the key to adapting the immune phenomenon of some animals to a vaccine capable of inducing broadly protective immunity in many animals lies in identifying conserved epitopes on surface proteins which are necessary for infection. 

While the purported targets of neutralization have been extensively discussed in recent reviews, it is worth noting that several epitopes on the membrane (M) protein, GP5, GP2, GP3, and GP4, have been shown, or implicated, to harbor neutralizing activity [114,116,118,119,120,121,122,123,124]. However, knocking out only CD163 in the pig is sufficient to render animals non-susceptible to PRRSV infection and replication [24,25,125]. It is proposed that following endocytosis, CD163 associates with the virus within the endosome, resulting in uncoating of the virus and the release of the viral genome into the cellular cytoplasm [126]. Since CD163 is necessary for viral infection and replication, the logical next step is to identify the conserved regions of viral surface proteins, most likely the minor glycoproteins (GP2, GP3, and GP4), that interact with CD163 [124,127]. 

### 4.2. Non-Neutralizing Antibody Response

Traditionally, the non-neutralizing antibody response to PRRSV has been considered useful only for its ability to identify if an animal had been exposed and seroconverted to virus. Indeed, there are many structural and non-structural proteins of PRRSV which make this possible through their ability to induce a robust humoral immune response [15,80,102]. However, recent research on other pathogens has shown that non-neutralizing antibodies may play a much larger role in immunity than was previously appreciated [128,129,130,131]. Alternative antibody functions, such as antibody dependent cell-mediated cytotoxicity (ADCC), antibody-dependent complement-mediated cytotoxicity (CDC), and antibody-dependent complement-mediated virolysis may be important in the clearance of virus and virally infected cells from an animal. To our knowledge, there are only two published papers investigating non-neutralizing antibody functions in the context of PRRSV infection [59,132]. Both of these in vitro studies utilized a PRRSV-1 virus and failed to find an effect of ADCC and CDC on infected cells. However, experiments focused on PRRSV-2 viruses with extended time points beyond 12 h are warranted. A more extensive review of non-neutralizing antibody functions can be found in the cited review [133]. 

## 5. The B Cell Response

If antibodies are the most important effectors of the immune system against viral infection, then B cells that make the antibodies are the most important cells. Previous research on the interaction between PRRSV and the porcine B cell is contradictory. It has recently been suggested that PRRSV infection results in lymphocyte apoptosis and immune impairment [61]. Several sources have shown that PRRSV largely or exclusively induces a specific humoral response to infection [51,134]. Other studies report that PRRSV infection results primarily in polyclonal B cell activation leading to hypergammaglobulinemia and the development of immune complexes [135,136,137,138]. The majority of work describing infection leading to polyclonal activation and hypergammaglobulinemia was performed in germ-free isolator piglets. This model is very effective for comparing B cell and antibody repertoire development in the fetus, as the germ-free status of the pigs removes many of the variables present when experiments are performed on conventionally reared animals [139]. However, these animals are deprived of the microflora and maternal antibodies to which conventional animals are exposed. As a result, the translation of immunological outcomes observed in isolator pigs to conventional pigs must be performed with caution. Studies in mice show that the immune systems of specific-pathogen free laboratory mice are similar to neonatal human immune systems, whereas feral mice displayed immune systems more comparable to adult humans. Effectively, the immune systems of germ-free animals may not display “normal” immune system phenotypes due to the lack of exposure to microflora [140,141]. 

**Figure 1:**
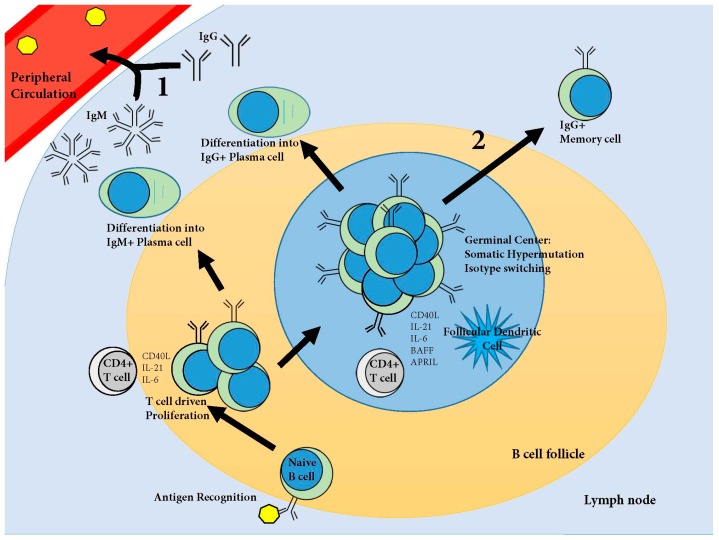
Development of systemic humoral immunity. Naive B cells move through the B cell follicles of the secondary lymphoid organs searching for antigens specific for their B cell receptors (BCR, surface immunoglobulin). Upon antigen recognition, the BCR is endocytosed, the antigen is degraded and then presented on the surface of the cell via Major Histocompatibility Complex (MHC)II. The B cell then migrates to the periphery of the B cell follicle searching for a Cluster of Differentiation (CD)4^+^ T cell specific for the same antigen. Upon T cell recognition of the MHCII presented antigen, the T cell stimulates the B cell by cytokine driven proliferation. The B cell proliferates and differentiates, some cells become immunoglobulin (Ig)M producing plasma cells, and other cells migrate into the B cell follicle where, with the help of cytokines from CD4^+^ follicular helper T cells and follicular dendritic cells, a germinal center is formed. In the germinal center, B cells proliferate and undergo somatic hypermutation and isotype switching. Affinity matured B cells then leave the germinal center as either IgG^+^ plasma cells or IgG^+^ memory cells. These cells constitute the first two lines of defense against reinfection: (1) affinity matured antibodies produced by plasma cells; and (2) memory cells which boost antibody titers upon antigen recognition. For an in depth review of this process based on data in humans and mice, please refer to Taylor et al. [143]. APRIL: a proliferation-inducing ligand; BAFF: B-cell-activating factor of the TNF family; IL: interleukin.

The development of protective humoral immunity, after vaccination or exposure to a pathogen, is dependent upon two lines of defense. The first immune defense is secreted antibodies, first from short-lived and then from long-lived, plasma cells residing somewhere in the body (Figure 1). The second line of defense is memory B cells (Figure 1). Memory cells are sentinels against reinfection which are activated upon antigen recognition to proliferate and differentiate into antibody secreting plasma cells, thus rapidly boosting circulating antibody titers with high affinity class switched antibodies [142]. 

Currently, there is scant research on the memory B cell response to PRRSV. Strong memory responses have been shown against nsp2, nsp7, N, and the 3′ end of GP5 [51,144]. The specific memory B cells are abundant in tonsil, lymph nodes draining the lungs and reproductive tract, and spleen. Unfortunately, there are many questions about the porcine memory response to PRRSV which have yet to be answered, including if memory cell kinetics closely mimic antibody kinetics, the response of PRRSV-specific memory pools upon homologous or heterologous viral challenge, and the importance of these cells in conferring protection against challenge. The development of sensitive and specific reagents, such as B cell tetramers, is a first step in being able to answer these critical questions. Additionally, it is possible that the key to understanding the broadly neutralizing response to PRRSV lies within circulating or lymphoid organ resident memory B cells. The potential to investigate these cells for identification of heavy and light chain antibody sequences is reviewed in Rahe and Murtaugh [133].

### Plasma Cells

Plasma cells are terminally differentiated B cells responsible for making antibodies. Apart from the immature plasmablast, two types of plasma cells have been defined in the mouse and human [145,146]. Short-lived plasma cells quickly boost antibody titers while long-lived plasma cells maintain circulating antibody titers in the face of continual antibody degradation. Mulupuri et al. identified PRRSV-specific plasma cells in several secondary lymphoid organs, such as the spleen, tonsil, sternal lymph node, and inguinal lymph node [51]. Interestingly, no PRRSV-specific or KLH-specific plasma cells were found in the bone marrow of immune pigs [51]. This was surprising, as the bone marrow has been long considered as the reservoir for long-lived plasma cells in both mice and humans [147,148,149]. It then begs the question, do pigs have long-lived plasma cells and, if so, where do they reside? Mulupuri et al. found PRRSV and KLH specific plasma cells in secondary lymphoid organs 120 days after inoculation [51]. However, these cells may not be “long lived” as the prolonged viremia of PRRSV may result in a somewhat continuous stimulation of memory B cells resulting in the appearance of this plasma cell population in secondary lymphoid organs. 

It seems unlikely that pigs do not have long lived plasma cells, as the half-life of porcine antibodies in serum is, on average, approximately nine days [150,151]. Therefore, without long lived plasma cells, pigs would quickly lose humoral protection as antibody titers waned. The identification of the anatomic location as well as the understanding of mechanisms for inducing a strong long lived plasma cell response may be important for future vaccine design as well as comprehending host–pathogen interactions. 

## 6. T Cell Response

Interestingly, even though neutralizing antibodies have historically garnered the majority of attention in PRRSV immunology, it is well-known that pigs readily control infection in the absence of neutralizing antibodies. Furthermore, viremia is reported in the presence of neutralizing antibodies [152,153]. Therefore, there must be other facets of the immune system which effectively function to control infection and eliminate PRRSV from the host. While some of this activity may be attributed to non-neutralizing functions of antibodies, the T cell response to infection demands further investigation. A recent PRRS immunity review summarized previous research on functional T cell subsets, and PRRSV epitope targets, as well as gaps in T cell immunity [11]. Here, we provide context for the understanding of novel results that have not been comprehensively reviewed.

Early research on the T cell response to PRRSV identified a large, transient decrease in the CD4^+^/CD8^+^ T cell ratio early, usually within the first week, in the course of infection [154]. The change in this ratio could have been due to a temporary loss of CD4^+^ cells through apoptosis or to an increase in CD8^+^ cells due to antigen-specific proliferation [154]. The importance of these findings for clearance of PRRSV or protection from infection were not known at the time, and other explanations, such as fluxes in cell populations between spleen, other lymphoid tissues, and blood could not be discounted. 

Experiments to address the helper T cell type 1/helper T cell type 2 (Th1/Th2) paradigm in the pig showed that PRRSV induced a strong Th1 response, as expected, identified in vivo by an increased expression of Th1-specification factor Tbx21(T-bet) in CD4^+^ cells [155]. However, the finding is at odds with previously reports indicating that PRRSV infection results in the production of IL-10, a cytokine classically associated with a Th2 phenotype. Similarly, monocyte-derived dendritic cells (Mo-DCs) infected with PRRSV down regulate swine leukocyte antigen (SLA)-I, SLA-II, CD40 and CD80 as well as promote IL-10 secretion over IL-12 secretion [156]. Delineation of the Th1/Th2 response to PRRSV, elucidation of Th1/Th2-specific cytokine markers in swine, as well as identifying associated cytokine responses of dendritic cells within secondary lymphoid organs where T cell proliferation and differentiation is most likely to occur, would help to resolve these outstanding questions [157]. 

The Th17 cell has classically been identified, in mouse and human, as playing an important role in extracellular bacterial immunity through the production of the pro-inflammatory cytokines, IL-17A, IL-17F, and IL-22 [158,159]. IL-17 producing Th17 cells are known to exist in the pig [160]. The importance of this T cell subset in the context of PRRSV infection has recently been investigated. A strain of Chinese highly pathogenic PRRSV (HP-PRRSV) appeared to suppress Th17 cells in the peripheral blood and lungs of pigs, resulting in an increased susceptibility to secondary bacterial infections [56]. Remarkably, the effect was PRRSV strain-specific, as a non-HP PRRSV strain failed to elicit the same response. Future research into the T cell response to PRRSV, especially with T cell tetramers and functional ELISPOTs, will be essential for the characterization of both CD4^+^ and CD8^+^ antigen specific T cells. Understanding how antigen-specific T cells interact with both infected and uninfected antigen presenting macrophages and dendritic cells will be helpful for advancing the field of PRRSV immunity.

## 7. Natural Killer Cell Response

The natural killer cell is an innate lymphoid cell which can have a profound impact on adaptive immunity, but is also able to induce an early and rapid innate response against pathogens through a variety of mechanisms. NK cells produce cytokines, such as IFNγ, show cytotoxic activity against infected cells not expressing MHCI, can induce dendritic cell maturation, and effect the destruction of infected cells in ADCC [161]. However, NK cells may deploy even more extensive and important functions in porcine immunity than are currently realized. 

An early clue that NK cells were involved in innate responses to PRRSV was a sharp peak in serum IFNγ shortly after infection [162]. The acute response was attributed to NK cells, as the result was deemed too early for a T cell response, and suggested that decreased viral burdens in the lung prior to humoral or T cell responses could be due to the function of NK cells. However, it is known that porcine macrophages are also capable of producing IFNγ in the presence of PRRSV infection [163,164]. Furthermore, PRRSV appears to suppress the NK cell response without significantly affecting NK cell numbers [165,166,167,168]. The cause of this suppression has yet to be determined, although viral proteins, rather than soluble factors from cells, may be responsible [59]. Potential roles of additional NK cell functions, such as ADCC, in PRRSV immunity are poorly understood [133]. 

## 8. Conclusions

PRRSV has tormented the health and wellbeing of swine worldwide since its discovery in the late 1980s. Unfortunately, after almost 30 years of research into the porcine immune response to PRRSV, there is still no effective means for inducing a broadly protective immune response at the herd level. The reasons for this failure are not completely known, but presumably include mechanisms by which the virus subverts the immune system. The ability of the virus to rapidly mutate while not losing fitness challenges the host immune system to keep pace. At the same time, infection of macrophages, a key player in immunoregulation, challenges both innate and adaptive immune cell mobilization as well as induction of a coordinated response that is needed for effective control and elimination of the virus. 

Fortunately, foundational advances in the understanding of viral pathogenesis and immunity are enabling more informative investigations. The identification of CD163 as the necessary and sufficient receptor for infection supports the implications of broadly neutralizing antibodies that a conserved target is present on all PRRSV. Understanding how PRRSV surface glycoproteins interact with CD163 should lead to the identification of conserved epitopes which are necessary for infection. If, as appears to be the case, there is only one conserved way into the cell, then there must be a conserved viral sequence, or structure, which enables viral entry. Furthermore, the knowledge that pigs eventually develop sterilizing immunity, if given enough time, supports the concept that conserved epitopes exist on the virus. Therefore, the study of mature animals, which have cleared the virus, may provide the key to understanding how the immune system eventually gets the upper hand on the virus and cures infection. 

Even with seminal advances in several aspects of the study of PRRSV, there remains much to be understood and clarified. Currently, the published literature presents conflicting views on many aspects of PRRSV adaptive immunity, especially related to T and B cell responses and the production, or inhibition, of cytokines in the face of infection. The continued development of antigen-specific reagents, of high sensitivity and specificity, is needed for understanding how the host responds to PRRSV infection. Furthermore, it is important that future PRRSV studies focus on the relevant host animal, the conventional pig. While the study of this outbred animal species is perhaps challenging at times, it affords the ability to study the host–pathogen interaction in the only species in which the virus naturally interacts. Additionally, knowledge gained about the immunology of conventional pigs will accelerate immunological elucidation of other pig–pathogen interactions.

In conclusion, PRRSV continues to be the most burdensome pathogen of pigs worldwide, due to its propensity for immune evasion and manipulation. However, the continued study of the porcine immune response to infection, with improved reagents and methods, will illuminate those aspects of the host–pathogen interaction that are now hidden. It is through these discoveries that the complex question that is PRRSV will finally be answered.
